# Interactions of the EGFR juxtamembrane domain with PIP_2_-containing lipid bilayers: Insights from multiscale molecular dynamics simulations^☆^

**DOI:** 10.1016/j.bbagen.2014.09.006

**Published:** 2015-05

**Authors:** Khairul Bariyyah Abd Halim, Heidi Koldsø, Mark S.P. Sansom

**Affiliations:** Department of Biochemistry, University of Oxford, South Parks Road, Oxford OX1 3QU, UK

**Keywords:** EGFR, Juxtamembrane domain, PIP_2_, Molecular dynamics, Lipid bilayer

## Abstract

**Background:**

The epidermal growth factor receptor (EGFR) is the best characterised member of the receptor tyrosine kinases, which play an important role in signalling across mammalian cell membranes. The EGFR juxtamembrane (JM) domain is involved in the mechanism of activation of the receptor, interacting with the anionic lipid phosphatidylinositol 4,5-bisphosphate (PIP_2_) in the intracellular leaflet of the cell membrane.

**Methods:**

Multiscale MD simulations were used to characterize PIP_2_–JM interactions. Simulations of the transmembrane helix plus JM region (TM–JM) dimer (PDB:2M20) in both PIP_2_-containing and PIP_2_-depleted lipid bilayer membranes revealed the interactions of the JM with PIP_2_ and other lipids.

**Results:**

PIP_2_ forms strong interactions with the basic residues in the R645–R647 motif of the JM domain resulting in clustering of PIP_2_ around the protein. This association of PIP_2_ and the JM domain aids stabilization of JM-A dimer away from the membrane. Mutation (R645N/R646N/R647N) or PIP_2_-depletion results in deformation of the JM-A dimer and changes in JM–membrane interactions.

**Conclusions:**

These simulations support the proposal that the positively charged residues at the start of the JM-A domain stabilize the JM-A helices in an orientation away from the membrane surface through binding to PIP_2_, thus promoting a conformation corresponding to an asymmetric (i.e. activated) kinase.

**General significance:**

This study indicates that MD simulations may be used to characterise JM/lipid interactions, thus helping to define their role in the mechanisms of receptor tyrosine kinases. This article is part of a Special Issue entitled Recent developments of molecular dynamics.

## Highlights

• EGFR transmembrane–juxtamembrane dimer was simulated in PIP_2_-containing bilayers.
• PIP_2_ forms favourable interactions with basic residues of the JM region.
• Mutation or PIP_2_ depletion changes juxtamembrane interactions with the bilayer.
• Simulations support interactions with anionic lipids in activation of the EGFR.

## 1 Introduction

The epidermal growth factor receptor (EGFR/ErbB1/HER1) is a member of the ErbB family of receptors, and is the best studied receptor tyrosine kinase (RTK) [1]. It has three homologs in humans, namely ErbB2/HER2, ErbB3/HER3 and ErbB4/HER4. EGFR is involved in regulation of key events associated with cell growth, differentiation and migration [2]. Overexpression of or mutations in EGFR is associated with various diseases, including cancer [3,4]. EGFR is a 170 kDa transmembrane glycoprotein, containing 1186 amino acids subdivided into a large extracellular region (the ectodomain), a single spanning transmembrane (TM) domain, an intracellular juxtamembrane (JM) region, a tyrosine kinase domain, and a C-terminal tail [1]. Ligand binding to the ectodomain induces conformational changes and activation of EGFR in an allosteric manner leading to formation of an asymmetric kinase domain dimer on the intracellular side of the membrane [5,6]. It is also known that ligand-induced activation of EGFR involves conformational changes within the ectodomain that are coupled to a specific TM interface, a reorientation of the JM domain relative to the bilayer, and formation of an asymmetric kinase domain dimer [5–7]. However, certain mechanistic details remain elusive, in particular the role of interactions of the lipid bilayer with the TM and JM domains.

The JM domain plays a crucial role in the activation of EGFR [5,8–13]. It was suggested that the asymmetric kinase domain dimer is stabilized by the participation of the JM domain [5,7]. The JM domain begins at the C-terminus of the TM domain extending from residues 645 to 682, referred to as JM-A (645–663) to JM-B (residues 664–682) [5]. The NMR structure of the TM–JM dimer [7] provides evidence that the JM-A forms an antiparallel helix dimer, consistent with previous studies [5,7,12]. The JM-B interacts with the kinase domain by forming a “juxtamembrane latch” at the asymmetric dimer interface [5]. Both JM-A and JM-B are critical for dimerization and activation of EGFR, as the receptor loses its activity when both JM-A and JM-B are removed [5]. The JM has been suggested to be able to decode the incoming signal from specific ligands via adoption of distinct conformations of the JM domain [12]. The observed negative cooperativity of the EGFR also requires the presence of the JM domain [11,14]. Furthermore, a recent mutagenesis study of the JM also showed that this region is critical in the transition from an inactive to an active dimer [15].

The JM-A segment contains three clusters of positively charged (Arg and Lys) residues that have been suggested to bind to negatively charged lipids [16]. These three clusters of positively charged residues are R645/R646/R647, R651/K652/R653, and R656/R657. The central portion of the JM-A segment also has hydrophobic residues in an *i*, *i* + 3, *i* + 4 pattern within the L_655_RRLL_659_ motif in an α-helical conformation [12,7]. The monomer/monomer interface of the JM-A dimer is formed by L655, L658 and L659, and it appears that the JM-A association occurs outside the membrane as judged by NMR-derived water accessibility of the JM residues [7]. The NMR structure of the TM–JM domain revealed a specific contact interface at the N-terminal motif of the TM domain that is compatible with a proper orientation of the intracellular JM-A domain by the LRRLL motif [7]. Thus, both the TM and the JM play a critical role to facilitate and stabilize the asymmetric kinase dimer crucial for EGFR activation [5,6].

In terms of anionic lipids within cell membranes, a key role is played by phosphatidylinositol bis-phosphate (PIP_2_), which comprises ~ 1% of the phospholipids in the plasma membrane [17]. PIP_2_ is known to regulate EGFR, and the JM domain of EGFR has been implicated in interactions with PIP_2_ [18] and Ca^2 +^/Calmodulin binding [19]. In a recent study, Michailidis et al. reported that EGFR is regulated by PIP_2_ via the positively charged residue clusters in the JM-A domain. They also demonstrated that mutation of the first three Arg/Lys within the JM to Asn abolishes PIP_2_ binding [16]. A recent study by Matsushita et al. of the closely related Neu TM–JM using solid-state NMR and fluorescent microscopy demonstrated that the TM–JM form tight interactions with anionic lipids and indicates that the JM domain is released from membrane upon ligand-induced activation [20]. It is therefore important to more fully characterize the nature of EGFR/PIP_2_ interactions.

Molecular dynamics (MD) simulations provide a computational route to detailed analysis of membrane protein–lipid interactions [21,22]. Previously published MD studies of the JM interaction with anionic lipids using long time scale atomistic MD simulations (AT-MD) in a simple POPC/POPS bilayer demonstrated an extensive interaction between the JM and membrane [23]. However, the interaction of the JM with PIP_2_ has not yet been extensively studied by MD simulations. Here, we study the association of the JM domain with the bilayer in both PIP_2_-containing and PIP_2_-depleted membranes, using a multi-scale approach, which includes both coarse-grained MD (CG-MD) and AT-MD simulations. These simulations indicate that the first three basic residues of the JM-A (R645, R646, and R647) strongly interact with PIP_2_ and that these interactions play a key role in stabilizing the JM-A helices on the cytosolic side of the bilayer, enabling a promoting JM-A association compatible with the active asymmetric kinase dimer. We are able for the first time to provide high-resolution insight into how PIP_2_ may impact on EGFR activation and stability, by showing that this lipid species is important both for JM stability and position relative to the membrane in addition to the transmembrane dimer interactions, all of which could be essential in stabilization of the active conformation of the receptor.

## 2 Methods

### 2.1 Coarse grained molecular dynamics simulations (CG-MD)

CG simulations were performed using GROMACS4.5.4 and AT simulations using GROMACS4.6 (www.gromac.org) [24,25]. The WT simulations were performed using the NMR structure of EGFR TM–JM (PDB:2M20) [7], from which the mutant structure (see below) was also generated using MODELLER [26]. Atomistic structures were converted to a coarse-grained (CG) representation compatible with the MARTINI force field (v2.1) for CG-MD simulations [27,28]. The TM–JM dimer was simulated with elastic network model (ENM) restraints *within* each monomer where a harmonic potential was applied to all pairs of backbone particles within a cut-off distance of 7 Å to maintain the secondary structure of the protein [29]. ENM restraints were not applied *between* the monomers, thus allowing dynamic repacking of the TM and JM helix dimers during the course of the simulations.

An initial 100 ns CG-MD simulation was performed to self-assemble the phospholipids (POPC) around the dimeric protein. POPS and PIP_2_ molecules were subsequently incorporated into the equilibrated POPC bilayer by exchanging them for POPC molecules; this procedure was implemented using an in house lipid exchange script [30]. The systems were energy minimized using the steepest descent method for 5,000 steps prior to performing unrestrained production run simulations. Simulations were performed at 300 K for the POPC bilayer and at 310 K for the POPC:POPS (90:10), and POPC:POPS:PIP_2_ (90:9:1) bilayers. The systems consist of 247 lipids in a simulation box of 100 × 100 × 120 Å^3^. The Berendsen barostat was used for pressure coupling in the CG-MD simulations with coupling constant, τ_p_ = 10 ps and a compressibility of 5 × 10^− 5^ bar^− 1^.

### 2.2 Atomistic molecular dynamics simulations (AT-MD)

To investigate in more detail the interactions of lipids with the TM–JM domain suggested by the CG-MD simulations, three replicates of a 100 ns AT simulation were performed starting from the final snapshots of the corresponding three replicate CG-MD simulations of the WT in a POPC:POPS:PIP_2_ bilayer. Each CG system was converted to an AT representation using a fragment-based approach [31]. The initial atomistic systems were energy minimized and equilibrated for 1 ns with the protein Cα atoms restrained (force constant = 10 kJ/mol/Å^2^) and subsequently followed by unrestrained production runs. The protein was simulated using the GROMOS 53a6 force field [32]. The LINCS algorithm was used to constrain bond lengths [33]. For the AT-MD simulations the particle mesh Ewald (PME) method was used to model long-range electrostatics [34]. A V-scale thermostat [35] was used for temperature coupling (temperature: 310 K) and the Parrinello–Rahman barostat [36] was used for semi-isotropic pressure coupling (pressure: 1 bar). Table 1 provides a summary of the simulations performed.

Simulation results were analysed using GROMACS tools and locally written codes. Visualization of the trajectories was performed in VMD [37]. Images were generated with VMD.

## 3 Results

### 3.1 Interactions of the JM region with PIP_2_

**Fig. 1:**
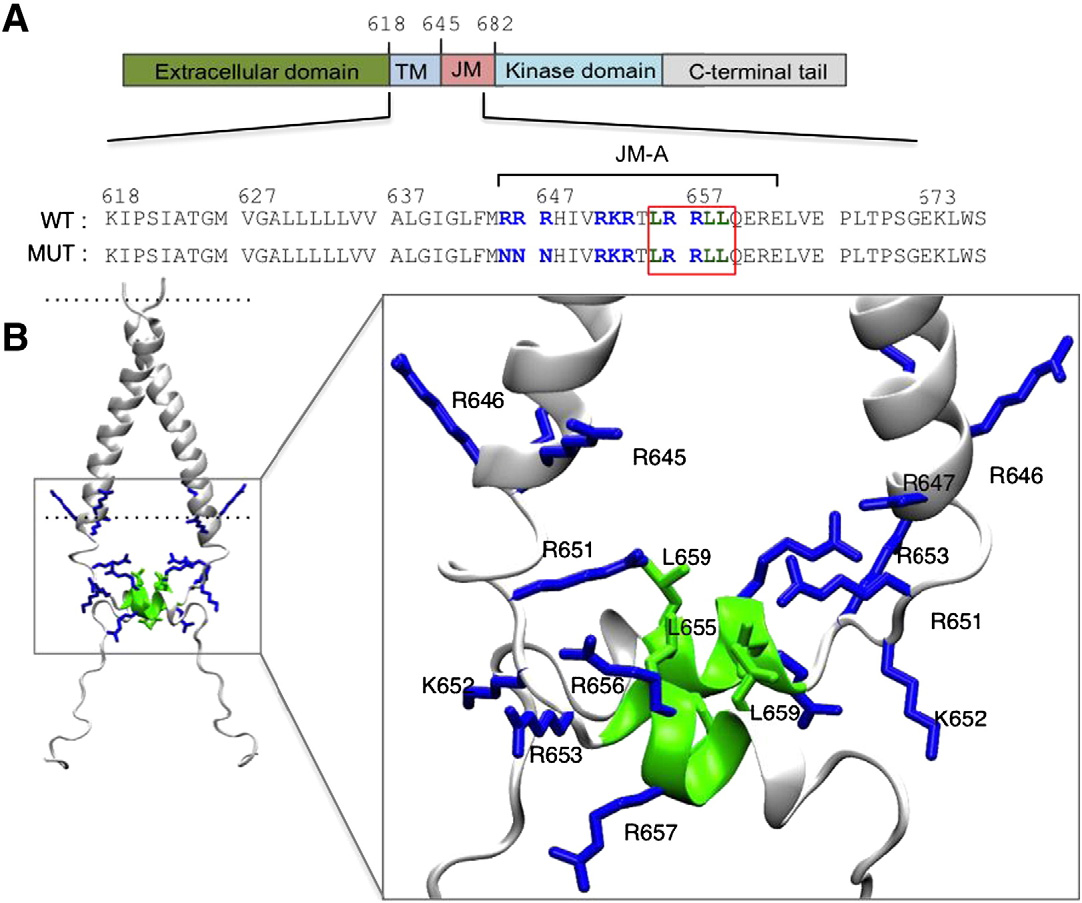
(A) Schematic representation of EGFR domains organization showing the location of the ectodomain, TM, JM, kinase and C-terminal domains (top). The sequences correspond to the TM–JM structure that was used in this study. The LRRLL motif (655–659) which forms the anti-parallel helix dimer of the JM-A (645–663) in the NMR structure is highlighted in the red box. (B) The NMR structure (PDB:2M20) of the TM–JM dimer with the lipid bilayer indicated as dotted lines. A zoom in of the JM domain focusing on the JM-A segment, the basic residues of the JM-A are shown as stick representation in blue and the LRRLL motif of the JM-A in green (right).

**Fig. 2:**
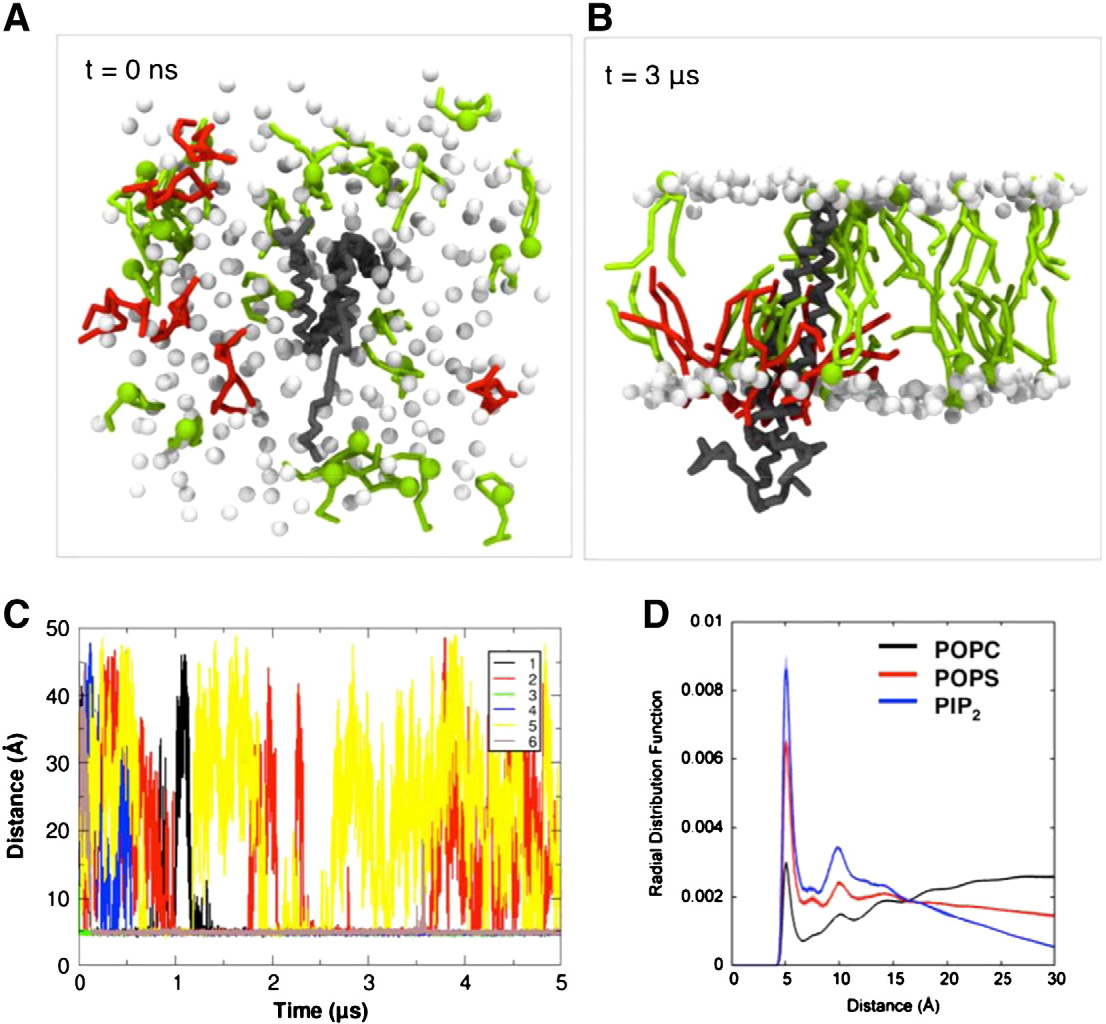
(A) A view from the cytoplasmic surface of the starting configuration at *t* = 0 ns in which the PIP_2_ molecules (red) are randomly distributed in the bilayer about the central protein (black/grey). (B) A snapshot of the system at *t* = 3 μs in which the PIP_2_ molecules can be seen to interact with the protein. POPS lipid molecules are shown in green, and the phosphate groups of the POPC molecules are shown as white spheres. The rest of the components of the system (water, ions, POPC lipid tails) are not shown for clarity. (C) The time evolution of minimum distances between PIP_2_ and protein for a representative CG-MD simulation of the TM–JM (WT) in a POPC:POPS:PIP_2_ bilayer. A different colour is used for each of the PIP_2_ molecules. (D) Normalized CG radial distribution function (RDF) for the different lipid species around the protein in a POPC:POPS:PIP_2_ bilayer. The POPC, POPS, and PIP_2_ are shown in black, red and blue respectively.

To explore the nature of interactions between the anionic lipids and the JM region of the EGFR TM–JM dimer, the NMR structure (PDB id 2 M20) was used as the starting point for CG-MD simulations in a POPC:POPS:PIP_2_ bilayer (Fig. 1; Table 1). The protein model consisted of residues 618 to 673 from the TM–JM sequence of the EGFR (sequence K^618^IPSIATGLVGALLLLLVVALGIGLFIRRRHIVRKRTLRRLLQERELVEPLTPSGE^673^KLWS) and thus included the TM helix dimer, the antiparallel JM-A helix dimer and part of the JM-B sequence (as a random coil) at its C-terminus (Fig. 1). The lipid composition was selected to provide a simplified model of the anionic lipids present within the inner leaflet of mammalian plasma membranes. The PIP_2_ molecules were randomly positioned in the inner leaflet of the bilayer (e.g. Fig. 2A) and three repeats of each simulation were performed. Over the course of the simulations these PIP_2_ molecules were observed to cluster around the membrane proximal section of the JM-A region (Fig. 2B). To monitor the interaction of the PIP_2_ molecules with the protein, we calculated the time dependent evolution of the minimum distance between the protein and the six PIP_2_ molecules over the course of the CG-MD simulations (Fig. 2C). Initial contacts between the PIP_2_ molecules and the protein were established within the first 0.5 μs. Three to four PIP_2_ molecules formed long-lived interactions with the proteins, whilst the other PIP_2_ molecules formed more transient interactions, and dissociated multiple times during the simulations. This suggests an approximate stoichiometry of 1 TM–JM dimer relative to 4 PIP_2_ molecules for the core complex.

**Fig. 3:**
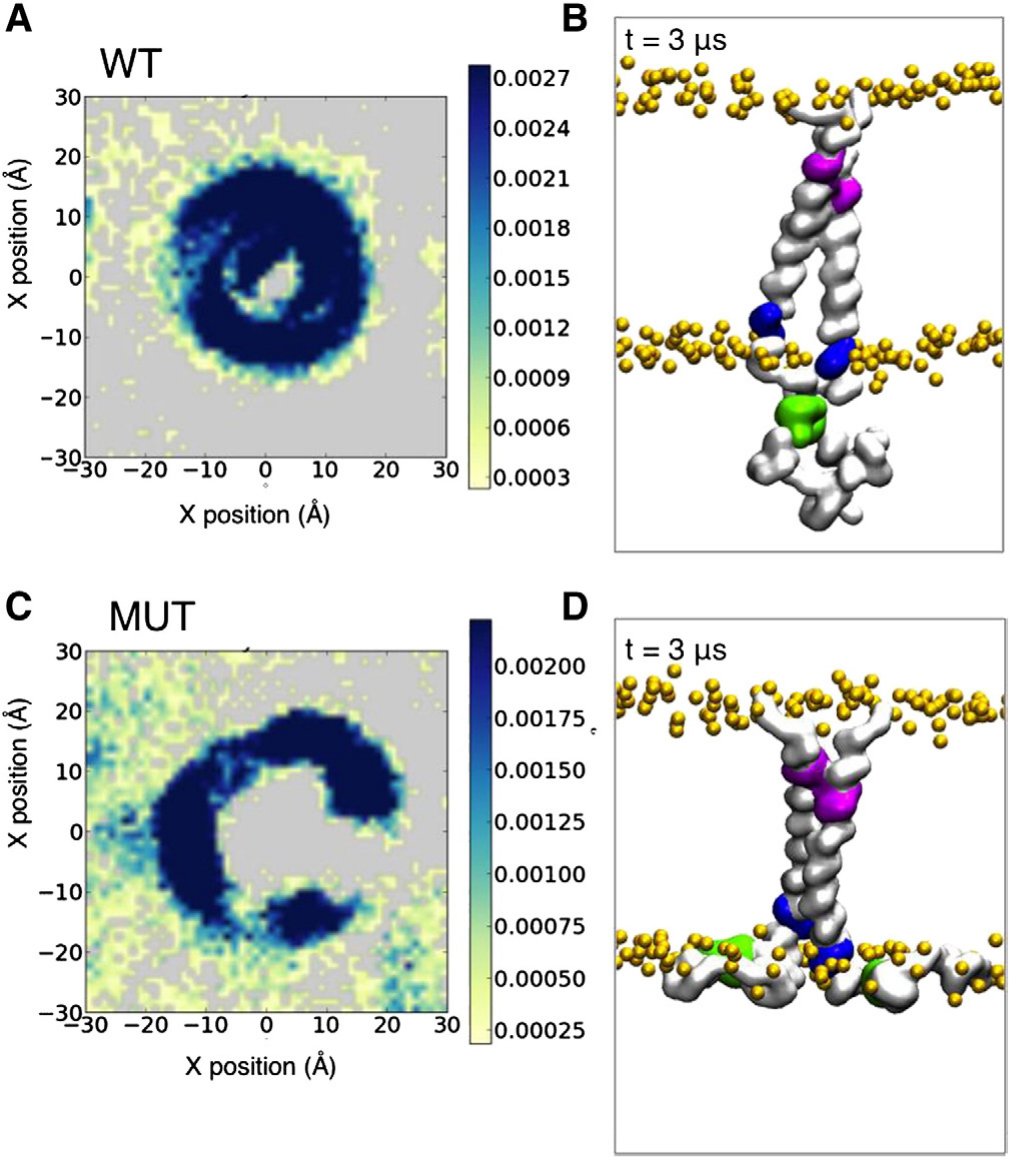
The time averaged 2D particle densities around the protein are used to illustrate clustering of anionic lipids around protein for the WT and the mutant TM–JM dimers. (A) Clustering of PIP_2_ in the lower leaflet of the POPC:POPS:PIP_2_ bilayer in the WT and (B) a snapshot of the final frame of a representative simulation. (C) Clustering of PIP_2_ in the lower leaflet of the TM–JM MUT (i.e. the R645N/R646N/R647N (ASN3) mutation). (D) A representative snapshot of the TM–JM conformation for the MUT dimer. The N-terminal motif (625–629), the first three JM residues 645–647 and LRRLL motif are shown as surface representation in magenta, blue and green respectively. The Cα trace of the protein is shown as surface representation in grey and the phosphate groups of the lipids are shown as orange spheres.

In order to compare the spatial distributions of the different lipid species around the TM–JM dimer, we calculated radial distribution functions (RDF) of all the lipids (Fig. 2D). From the RDFs it can be seen that the probability of observing PIP_2_ in the first shell around the protein (i.e. at a radial distance between ca. 4 and 6 Å) is much higher than for either POPS (anionic) or POPC (zwitterionic). This suggests that the interaction between the EGFR TM–JM and lipids is dominated by PIP_2_. Examining their probability of occurrence projected onto the bilayer plane reveals the PIP_2_ molecules to be clustered around the protein in a ring-like pattern. Thus there is a clear local segregation of lipids whereby PIP_2_ is confined to the membrane region in close proximity to the membrane proximal JM residues of the protein (Fig. 3A).

To assess the stability of the TM helix packing within the TM–JM dimer, we evaluated the helix crossing angle distribution and the inter-protein contact matrix (Supplementary Material Figs. S1A and S2A), averaging these across all three simulations. Both the crossing angle and inter-protein contacts remained stable throughout all three simulations. The main crossing angle was ca. − 30° (compared to ca. − 40° in the NMR structure), and the helix–helix packing was mediated by the N-terminal TM helix T624-G625-x-x-G628-A629 sequence motif, again as in the NMR structure. We note that this packing mode of the TM helices is as seen in earlier CG-MD simulations of a dimer of the TM helix alone (in DPPC bilayers) [38]. This suggests that in the presence of PIP_2_ the JM region interactions (both protein–lipid and protein–protein) stabilize the preferred mode of packing of the TM helices.

### 3.2 Mutations of the membrane proximal JM-A region

In a study of lipid interactions with peptides corresponding to the JM domain alongside effects on EGFR signalling, Michailidis et al. showed that neutralization of three positively charged residues in the membrane proximal region of JM-A (i.e. R645, R646 and R647) by mutation to asparagine (termed the ASN3 mutation in reference [16]) abolished EGFR phosphorylation. We therefore repeated our simulations using a model of the ASN3 mutant TM–JM dimer based on the NMR structure of the wild type (WT) dimer. Three significant differences between the WT and the ASN3 mutant were observed. Firstly, in the ASN3 mutant there was a change in the PIP_2_ distribution relative to that for the WT (Fig. 3C) such that the PIP_2_ molecules formed a wider and somewhat more diffuse ring around the central protein. Secondly, the conformation of the JM region changed, disrupting the anti-parallel JM-A helix dimer and so as to allow the remaining cationic residues of the JM region to ‘spread’ over the surface of the lipid bilayer (Fig. 3D). Thus, in the simulations of the ASN3 mutant, the JM domain makes contacts with the lipid bilayer by either one or both JM-A monomers. Consequently, the mutant TM–JM dimer exhibits changes in PIP_2_ lipid clustering and in the nature of the interactions of the JM region both within the dimer and with the bilayer.

These changes in the JM–membrane interactions for the ASN3 mutant influence the packing of the TM helices. Thus, analysis of the crossing angle distribution (Supplementary Material Fig. S1B) suggests a loosening of the interactions of the TM helices, resulting in a mixture of right-handed and left-handed helix packing (as evidenced by a bimodal average distribution, as in one of the simulations the packing switched from right-handed to left-handed). Also the average inter-protein interactions (Supplementary Material Fig. S2B) revealed loss of the JM–JM interactions in addition to a weakening of the TM–TM helix interactions compared to the WT simulations. This destabilization of the dimer and of the position of the JM away from the membrane in addition to the destabilization of the membrane-distant position JM-A helices in the ASN3 mutation indicates that the favourable interactions between PIP_2_ and R645, R646 and R647 are necessary to maintain an active state of the receptor.

**Fig. 4:**
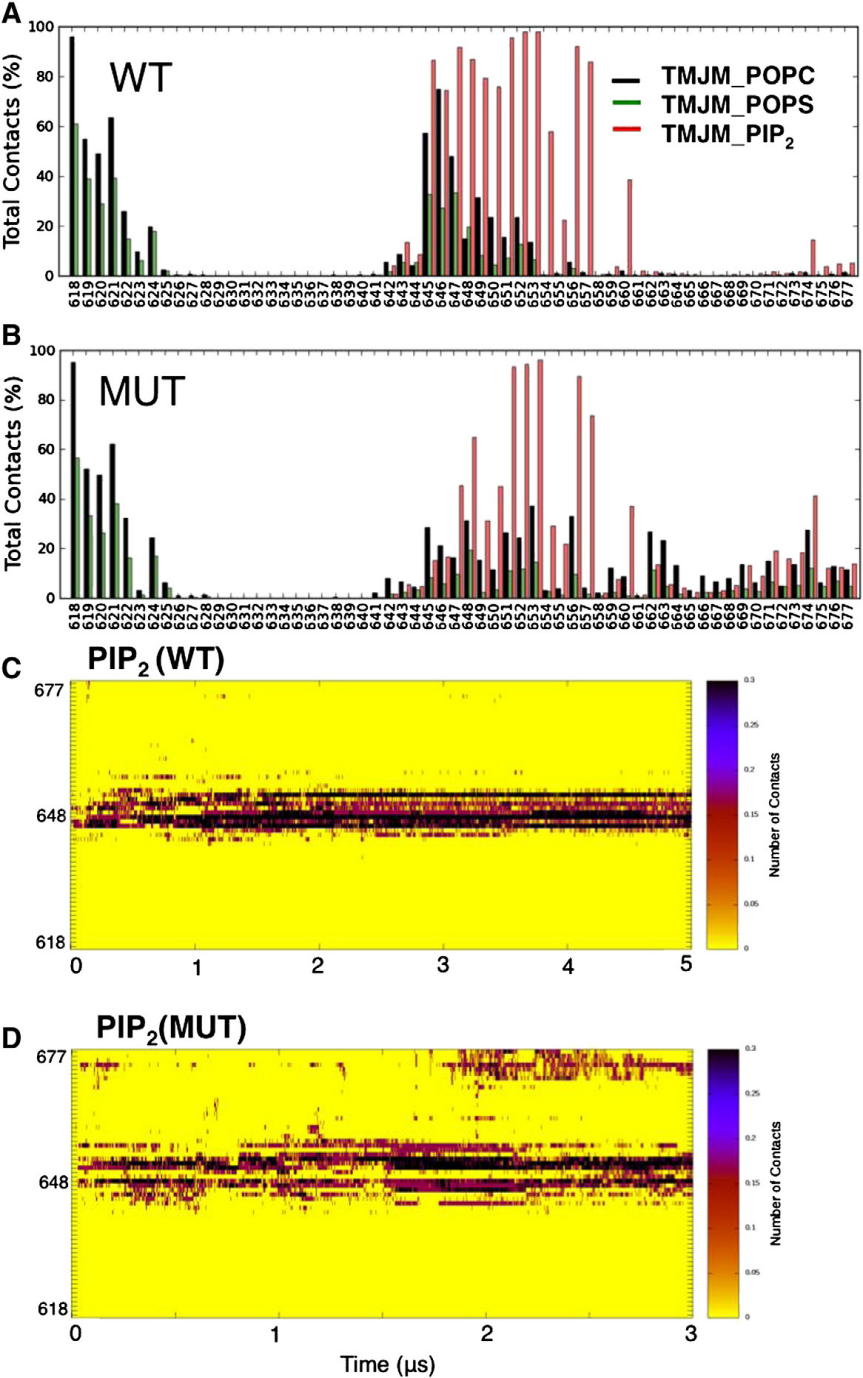
Normalized average number of contacts (within a 6 Å cut-off) between the phosphate groups of the POPC, POPS and PIP_2_ lipids and protein for the (A) WT and (B) mutant TM–JM dimers. The POPC, POPS and PIP_2_ lipids are shown as bars in black, green and red respectively. The interactions between the head group of the lipids and protein within 6 Å cut-off calculated over the course of the simulations for a representative CG-MD simulations of the (C) WT and (D) the mutant in the POPC:POPS:PIP_2_ bilayer.

To identify the key residues involve in the protein–lipid interactions during the CG-MD simulations, average protein–lipid contacts were calculated. This analysis confirmed that the positively charged residues in the membrane proximal JM-A region (i.e. residues 645–657) are involved in interactions with PIP_2_ for more than 50% of the total contacts, suggesting strong interactions between the basic residues and PIP_2_ (Fig. 4A,C). Significant contacts to PIP_2_ were also observed for the other positively charged residues (651–653, 656 and 657). As anticipated, the ASN3 mutation resulted in loss of the interactions of residues 645–647, but the more membrane distal cationic residues of JM-A retained their contacts to PIP_2_ (Fig. 4B, D). This analysis confirms that in the ASN3 mutation the disruption of the JM-A position allows extensive interactions between PIP_2_ molecules and the R651/K652/R653 and R656/R657 and other more distal (e.g. K674) residues along the JM segment.

### 3.3 Simulations in PIP_2_-depleted membranes

CG-MD simulations were carried out in membranes without PIP_2_, both in POPC and in POPC:POPS bilayers (Table 1), using the same protein structures as in the previous PIP_2_-containing simulations. In the POPC:POPS bilayer, a degree of clustering of POPS molecules was observed around the protein, forming either the ring-like pattern observed for PIP_2_ or two or three discrete patches around the protein in the inner leaflet. POPS lipids also clustered in the outer leaflet of the bilayer around the TM proximal residues 618 to 624, with more than 50% of total contacts with K618. In both the simulations in POPC and in POPC:POPS bilayers, the JM region made some interactions with the membrane, forming an average of 10–20% of total contacts with the bilayer.

**Fig. 5:**
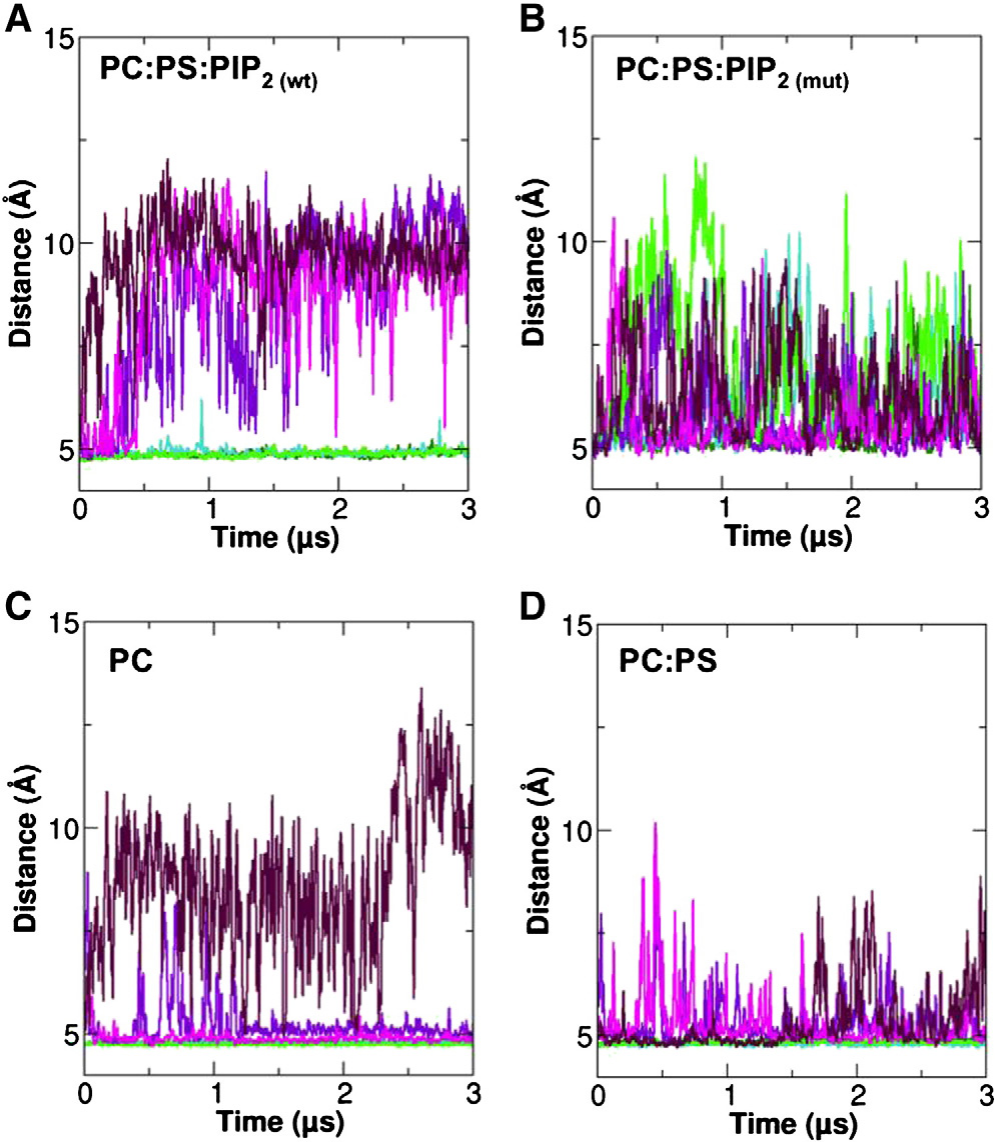
Comparison of the evolution of minimum distances between the COM of the R645–647 motif (green) and of the COM of the LRRLL motif (purple) relative to the COM of the phosphate groups of the cytoplasmic leaflet of the bilayer. The distances were calculated for the (A) WT and (B) the mutant dimer in a POPC:POPS:PIP_2_ bilayer, and for the WT dimer in (C) a POPC bilayer and a (D) POPC:POPS bilayer. Individual CG-MD simulation of the three repeats in all of the bilayer types is shown in different shades of green and purple for R645–647 and LRRLL region respectively.

The interactions of the JM region with bilayers as a function of lipid composition can be analysed via comparison of minimum distances of the COM of the membrane proximal residues R645–647 or of the COM of the LRRLL motif from the COM of the phosphate groups of the inner leaflet of the bilayer (Fig. 5). For the WT TM–JM dimer in the POPC:POPS:PIP_2_ bilayer, the R645–647 residues remain close to the bilayer surface, whilst the LRRLL motif of the antiparallel JM helix dimer remains more distant from the bilayer surface, even if when the simulations were extended to 5 μs (Supplementary Material Fig. S3). As seen previously, for the same PIP_2_-containing bilayer the ASN3 mutant shows a much looser interaction of the R645–R647 region with the bilayer, and also the LRRLL motif transiently contacts the bilayer surface. The average distance of association of the JM motifs from the bilayer may also be seen by comparing the density profiles for the R645–647 and LRRLL motifs relative to the lipid phosphate groups of the lipid bilayer (Supplementary Material Fig. S4). Overall, this comparison clearly demonstrates that the interaction of the R645–647 motif with the PIP_2_ molecules in the bilayer is required to stabilize the antiparallel LRRLL motif-containing helix dimer at its location slightly displaced relative to the intracellular lipid bilayer surface.

### 3.4 Atomistic simulations

**Fig. 6:**
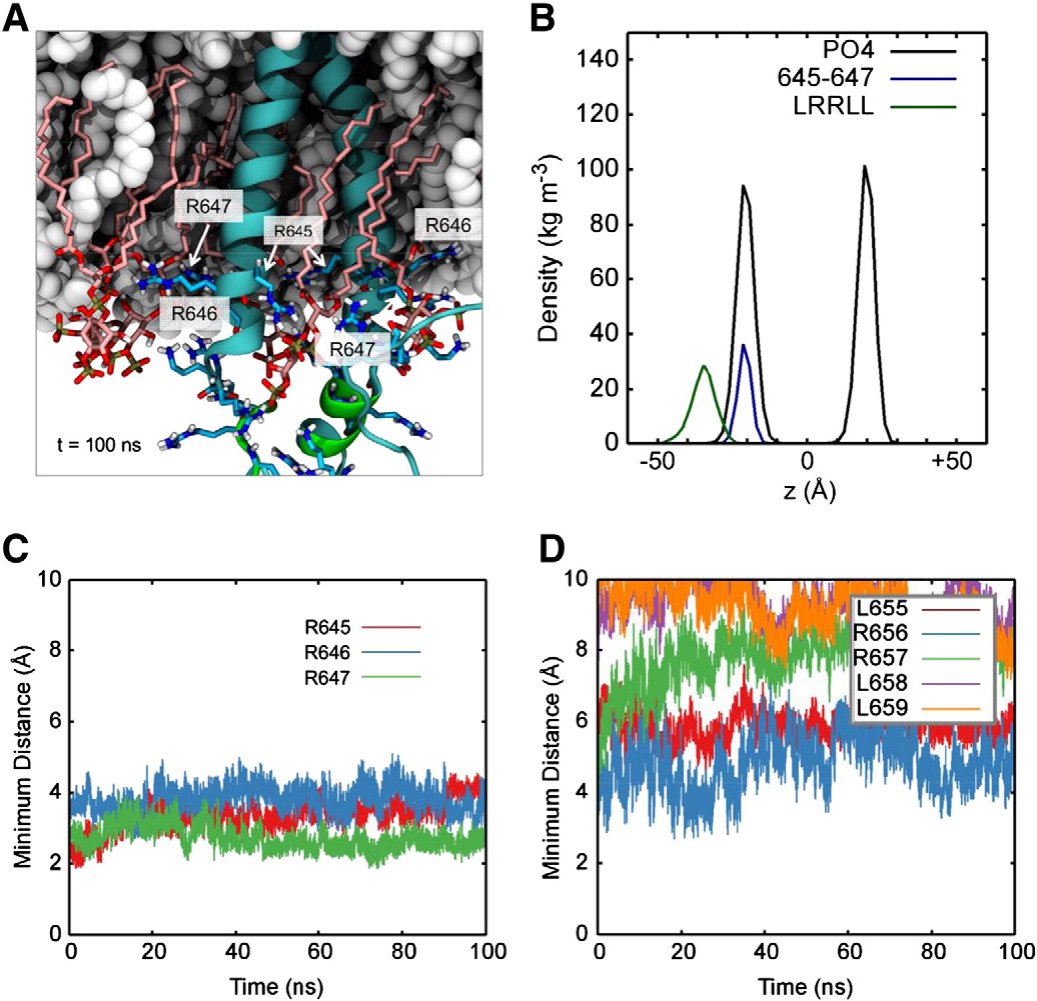
(A) Snapshot of the AT-MD simulation of the EGFR TM–JM dimer at *t* = 0.1 μs, zooming in on the JM region. The protein is shown as cyan cartoon representation with the LRRLL motif in green. The POPC and POPS lipids are shown as white spheres and PIP_2_ lipids are shown in stick representation with the carbon in pink. Arginine and lysine side chains are shown in sticks with cyan carbon atoms. The side chains of the R645–647 motif are highlighted with labels. (B) Density profiles along the membrane normal for the R645–647 motif (blue) and the LRRLL motif (green) relative to the positions of the lipid phosphates (black), averaged over three repeats of the AT simulations. (C) The minimum distance between the first cluster of positively charged residues (R645/R646/R647) and PIP_2_, averaged over both monomers and all three simulations. (D) The minimum distance between residue 654–559 (which contains the LRRLL motif) and PIP_2_ over the course of the simulations, again averaged over both monomers and all three simulations.

To refine the models, the final snapshots of the TM–JM dimers predicted in the three repeat CG-MD simulations in the POPC:POPS:PIP_2_ bilayer were converted to atomistic (AT) representations, followed by short (0.1 μs) atomistic simulations. Such simulations allow the protein/lipid interactions to relax but do not permit major rearrangements of e.g. the PIP_2_ clusters (Fig. 6A). Conformational drift of the TM domain during the AT simulation was assessed by measuring the Cα RMSD values of the transmembrane helices (Pro620–Met644) relative to the initial structure (Supplementary Material Fig. S5). The RMSD of the TM domain showed that the AT system reached equilibration within 45 ns to approximately 3 Å, which is expected based on an AT system obtained from a CG simulation [31]. The LRRLL motif within the JM-A region remained largely helical and did not attach to the bilayer. In all the AT simulations, the LRRLL motif remained unbound to the membrane, as illustrated in the density profiles (Fig. 6B). The distance between the membrane and the LRRLL motif was similar between the CG and AT simulations further validating the application of CG-MD.

Analysis of the closest approach distances (Fig. 6C) confirms tight interactions (2 to 3 Å) between the sidechains of R645–647 throughout the AT simulations and the LRRLL motif (residues 655 to 659) sits more distantly still (> 4 Å) from the bilayer (Fig. 6D). The interactions between these arginine residues and PIP_2_ both included interactions with the phosphate and hydroxyl groups, and the arginine side chains were observed to point towards the PIP_2_ head groups (Fig. 6A).

## 4 Discussion and conclusion

CG-MD simulations of the JM domain of EGFR in bilayers containing anionic lipids have revealed that the positively charged residues within the JM-A domain are able to attract and sequester PIP_2,_ resulting in clustering of this lipid species around the protein. The PIP_2_ molecules interacted favourably and extensively with the positively charged residues of the JM, and in particular with the R645–R647 cationic cluster, in good agreement with the experimental studies of Michailidis et al. [16]. Comparison of the interactions between the JM region and PIP_2_ in the CG-MD simulations of the WT and mutant (ASN3) suggests that the mutation not only reduces PIP_2_ binding but also results in destabilization of the antiparallel JM helix dimer and less tight packing of the transmembrane helices. This in turn resulted in more extensive interactions between the more C-terminal residues of the JM region and the membrane. Significantly, in PIP_2_ depleted membranes, the JM-A dimer also was destabilized and interacted with the membrane.

Refinement of the model from the WT CG-MD simulation via atomistic simulations in a POPC:POPS:PIP_2_ bilayer confirmed that the TM–JM dimer was stable. This is also in agreement with the behaviour of the JM domain of a closely related protein, the Neu (rat ErbB2) in a solid-state NMR and fluorescent microscopy study by Matsushita et al. [20]. They showed that the JM of the Neu TM–JM dimer was detached from the membrane in the presence of PIP_2_ [20]. Our simulations are also consistent with a long timescale all atom MD simulation by Arkhipov et al. [23] with anionic lipids. Although they did not include PIP_2_, they demonstrated that increasing amount of anionic lipid (POPS) stabilized the TM domain association at the N-terminal motif which favoured the antiparallel helix dimer of the JM-A segment. Our simulations demonstrate that PIP_2_ assists to further stabilize this TM–JM conformation. Taken together, these simulation and experimental studies support the notion that the positively charged residues of the JM-A domain are able to sequester PIP_2_, thus stabilizing the JM-A helices away from the membrane surface and so promoting a conformation corresponding to an asymmetric (i.e. activated) kinase.

It is important to consider the likely limitations of the simulation methods used. Firstly, CG-MD methods provide an approximation to more detailed, all-atom treatments of protein–lipid interactions. They allow long timescales to be readily addressed, but approximate the nature of interactions, especially in terms of electrostatics and H-bonds. However, comparisons with experiment have shown that this approach can successfully predict the binding of e.g. PIP_2_ to Kir channels [21] and cardiolipin to cytochrome bc1 [39], and simulations have also been used to explore anionic lipid interactions with juxtamembrane regions of other receptors [40]. We are therefore reasonably confident that combining CG-MD with AT-MD simulations provides meaningful insights into PIP_2_–receptor interactions. However, future simulations will need to focus on both the complete EGFR protein dimer and also more complex mixtures of lipids present in cellular membranes.

In summary, we have demonstrated that PIP_2_ forms strong interactions with the JM domain and that the TM–JM-A helices are positioned distant from the membrane. This highlights that the interplay of the (anionic) lipid composition and the basic clusters of the JM region is critical to the orientation of the JM regions, as seen also in a number of experiments [5,7,12]. Additionally the transmembrane dimer stability is dictated by the interactions between the JM region and PIP_2_ indicating a more global role in the interactions between PIP_2_ and the initial three basic (R645, R646 and R647) within the JM. We have also shown selective interactions of the TM–JM dimer with PIP_2_ over POPS, further emphasizing the need for more complex lipid bilayer models in simulations of membrane protein dynamics and function.

## Figures and Tables

**Table 1:** Summary of simulations of the JM-TM dimer in PIP_2_-containing and PIP_2_-depleted membranes.

Protein	Lipid bilayer composition	Duration of simulations
Coarse-grained		
TM–JM (WT)	POPC	3 × 5 μs
TM–JM (WT)	POPC, POPS (90:10)	3 × 3 μs
TM–JM (WT)	POPC, POPS, PIP_2_ (90:9:1)	3 × 5 μs
TM–JM_ASN3^a^	POPC, POPS, PIP_2_ (90:9:1)	3 × 3 μs
Atomistic		
TM–JM (WT)	POPC, POPS, PIP_2_ (90:9:1)	3 × 0.1 μs

a ASN3 is the R645N/R646N/R647N triple mutation in reference [16].
