# Supplementary material for: Interactions of the EGFR juxtamembrane domain with PIP2-containing lipid bilayers: Insights from multiscale molecular dynamics simulations
Source: Biochim Biophys Acta. 2015 May;1850(5):1017–25. doi: 10.1016/j.bbagen.2014.09.006 (PMC4547087; doi:10.1016/j.bbagen.2014.09.006)
Supplement: Supplementary file 1 — Supplementary figures. [file mmc1.docx]

**SUPPLEMENTARY MATERIAL for:**

**Interactions of the EGFR Juxtamembrane Domain with PIP_2_-Containing Lipid Bilayers: Insights from Multiscale Molecular Dynamics Simulations**

*Khairul Bariyyah Abd Halim, Heidi Koldsø, & Mark S P Sansom*


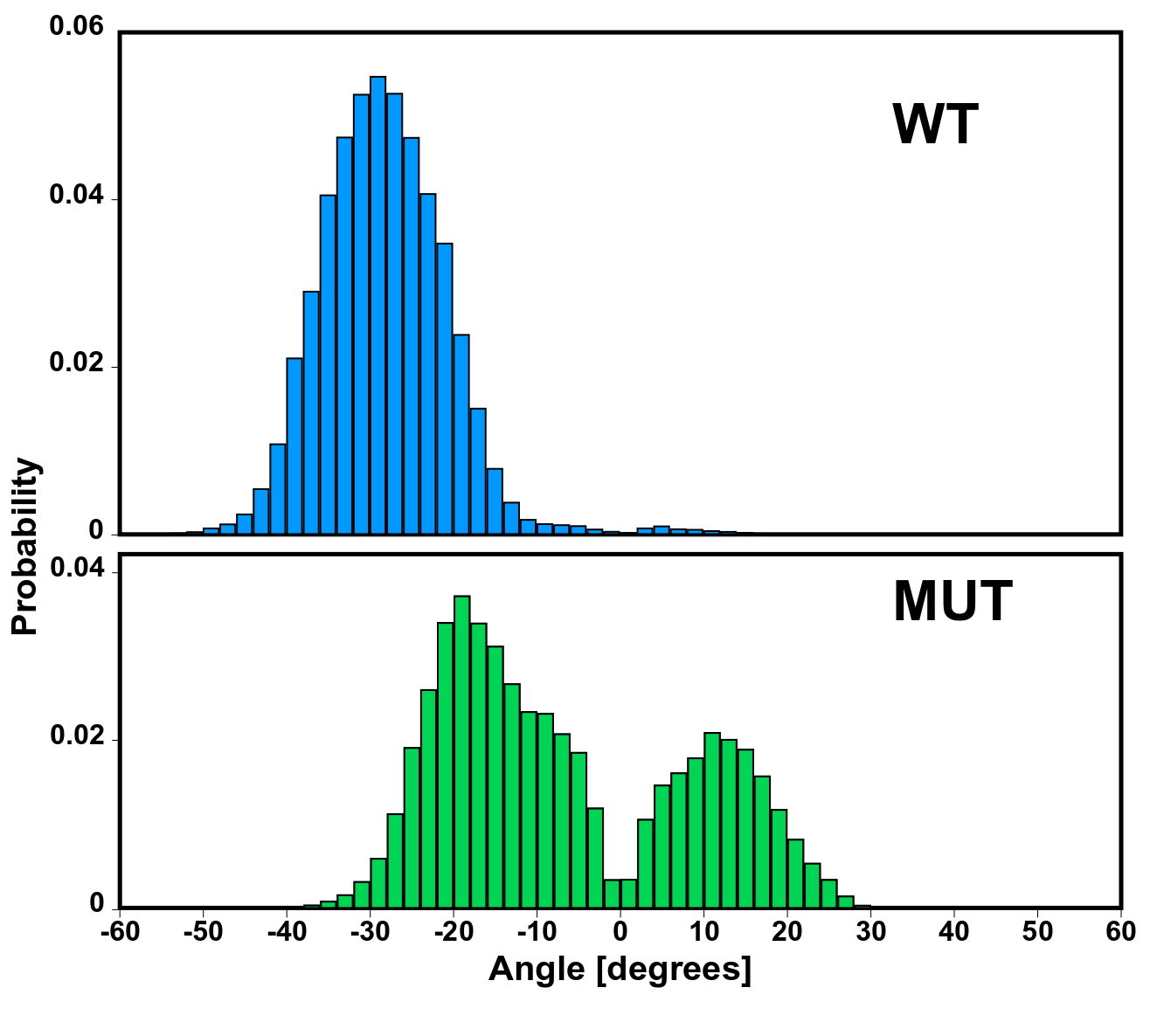


**B**

**A**

*Figure S1:*

Crossing angle distributions combined for all three simulations in POPC:POPS:PIP_2_ of (**A**) the WT and (**B**) the ASN3 mutant TM-JM helix dimers. Crossing angles were calculated between the two TM helices for timeframes when their centre of mass separation was within 10 Å.


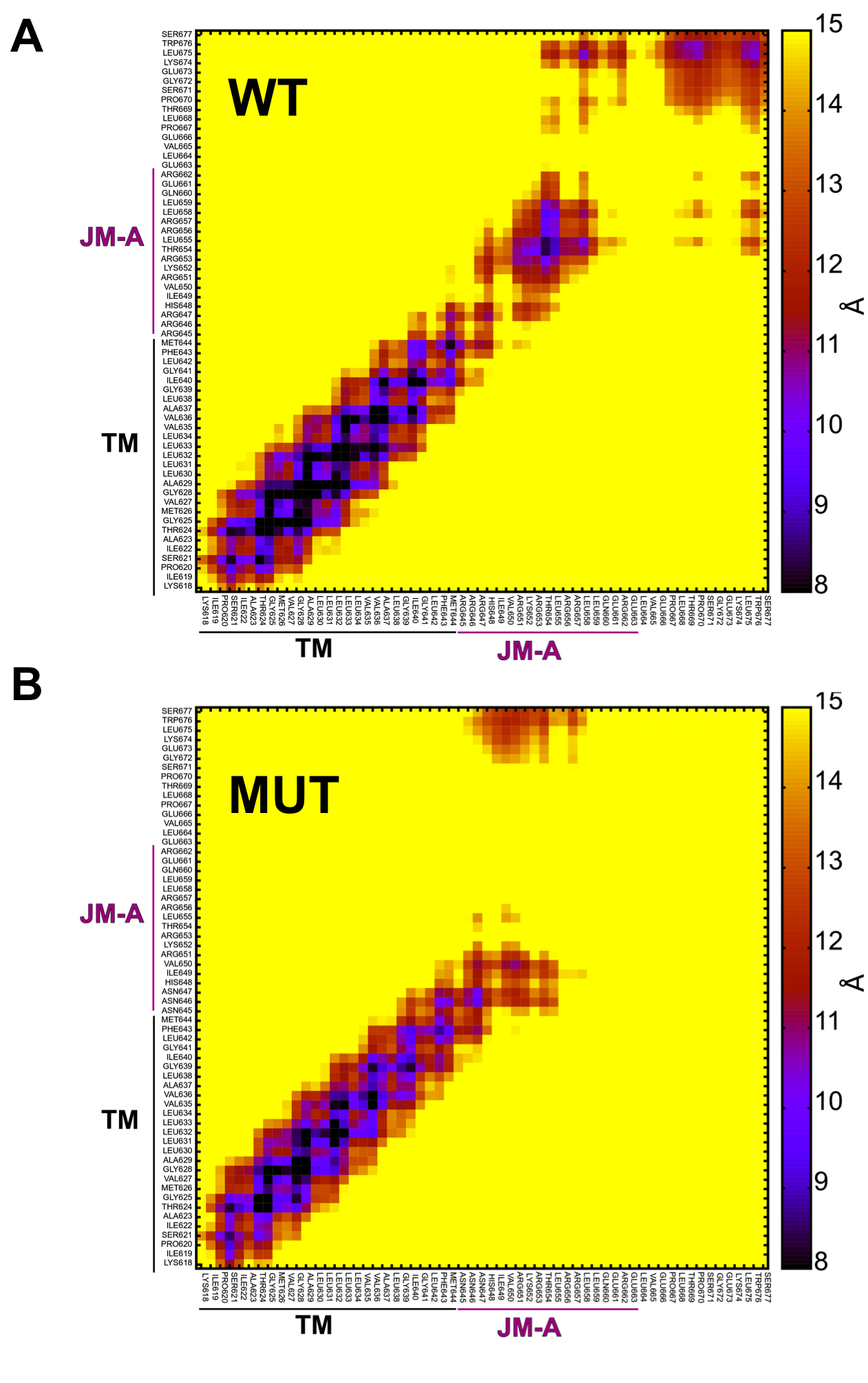


*Figure S2:*

Protein-protein distance contact matrix. The average distance has been calculated over all three simulations of (**A**) the WT and (**B**) the ASN3 mutant TM-JM simulations in POPC:POPS:PIP_2_.


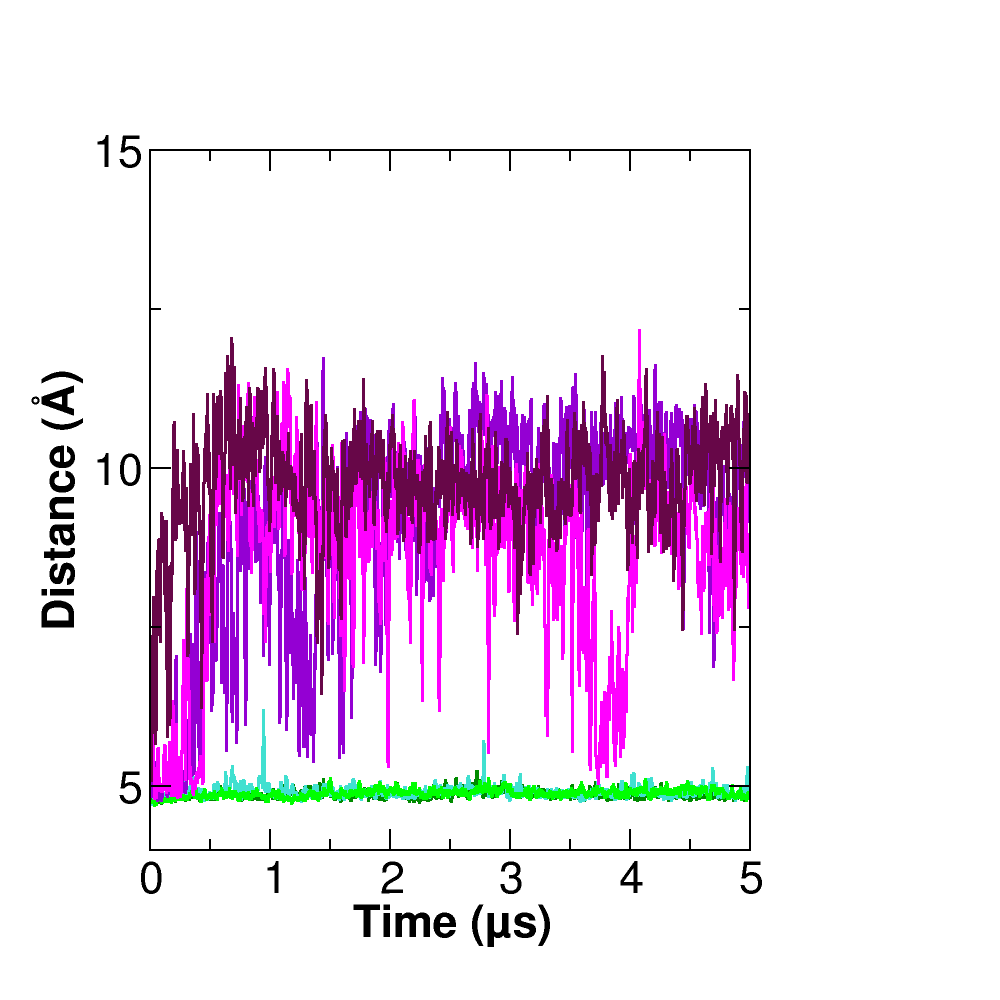


*Figure S3:*

The CG-MD simulations for TM-JM (WT) dimer in a POPC:POPS:PIP_2_ bilayer were extended to 5 µs. The distances between COM of the R645-R647 basic region to the COM of the cytoplasmic leaflet phosphate groups of the bilayer are in green, and between the COM of the LRRLL motif to the COM of the phosphates are shown as magenta. Different shades of magenta and green represent the three simulation repeats.


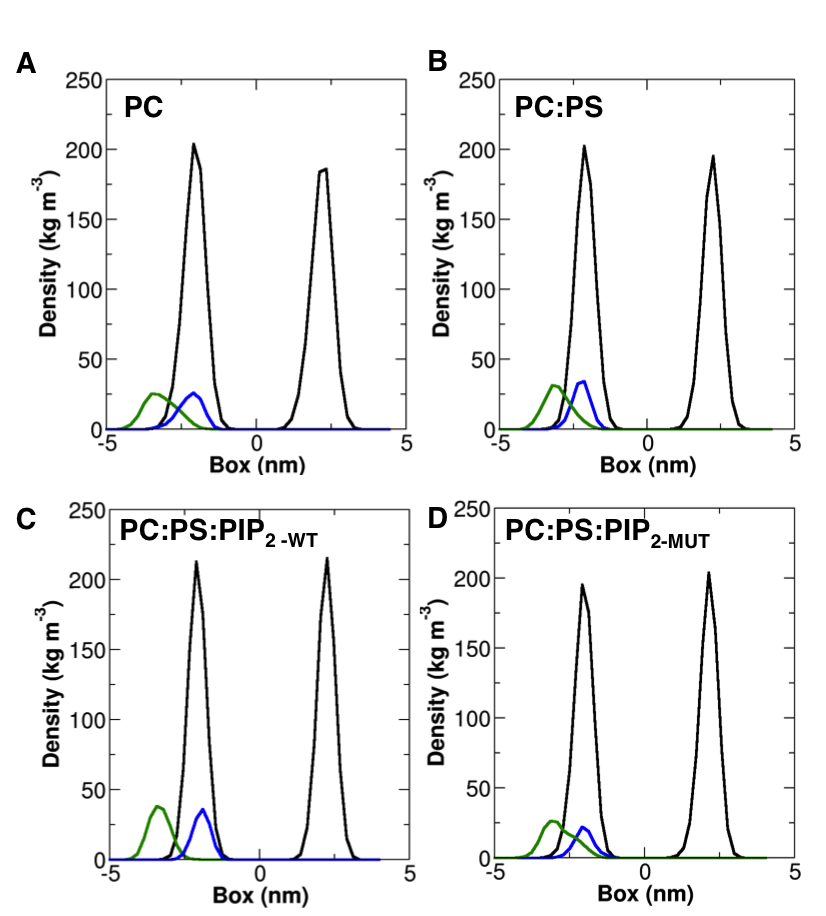


*Figure S4:*

Density profiles along the membrane normal calculated for the three WT simulations, (**A**) POPC, (**B**) POPC:POPS, and (**C**) POPC:POPS:PIP_2_, and for the (**D**) and ASN3 mutant POPC:POPS:PIP_2_ simulation. The R645/R646/R647 at the membrane proximal end of the JM-A region is in blue, the LRRLL motif in green, and the lipid phosphate groups in black.


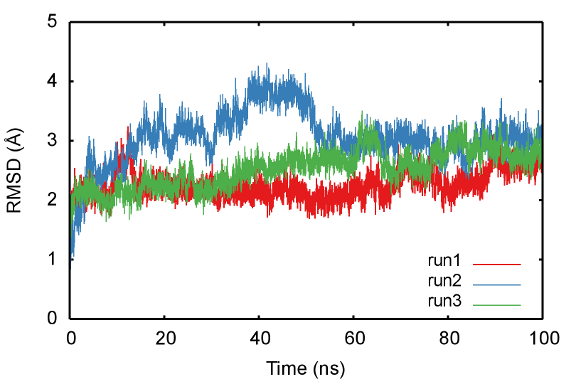


*Figure S5:*

Cα RMSDs for transmembrane domain residues as a function of time for the AT-MD simulations of the WT of the TM-JM dimer in a POPC:POPS:PIP_2_ bilayer.
